# Efficacy and safety of brolucizumab versus aflibercept in patients with neovascular age-related macular degeneration: a randomized trial in Indian patients

**DOI:** 10.1186/s40942-022-00401-4

**Published:** 2022-07-28

**Authors:** Sanjay Kumar Mishra, Pradeep Kumar, Srishti Khullar, Amrita Joshi, Alok Sati, Sonali Vinay Kumar, Deepesh Unni, Atul Kumar

**Affiliations:** Department of Ophthalmology, Army Hospital Research Referral, Delhi Cantt, Delhi, 110010 New Delhi India

**Keywords:** Neovascular age-related macular degeneration, nAMD, Brolucizumab, Aflibercept, VEGF, Indian

## Abstract

**Background:**

The current standard treatment for neovascular age-related macular degeneration (nAMD) involves intravitreal injections of anti-vascular endothelial growth factor (anti-VEGF) agents. The aim of the present study was to compare the effectiveness and safety of two anti-VEGF drugs: brolucizumab and aflibercept, in treatment-naïve nAMD Indian patients over a period of 48 weeks.

**Methods:**

A prospective, randomized, single-centre, single-blinded, two-arm comparative study was conducted between March 2021 and February 2022. Of the 114 patients, 56 received intravitreal injections of brolucizumab (6 mg/50 µL) while 58 received aflibercept (2 mg/50 µL). The patients received 03 initial loading doses at 4-week intervals of both the agents and then respective therapies were given as individualized *pro re nata* (PRN) regimen based on the signs of active macular neovascularization. The functional and anatomical outcomes measured were mean change in best-corrected visual acuity (BCVA, logMAR), central macular thickness (CMT, µm), presence of intraretinal fluid, subretinal fluid or subretinal hyper-reflective material. Furthermore, the average number of additional injections required after the loading doses, the injection-free interval and safety of both the drugs were also assessed.

**Results:**

Brolucizumab was found to be non-inferior to aflibercept in terms of mean change in BCVA (−0.13 ± 0.21 logMAR vs. −0.10 ± 0.15 logMAR) and reduction in CMT (−112.59 ± 81.23 µm vs. −86.38 ± 71.82 µm). The percentage of eyes with IRF and SHRM was comparable between both the groups while fewer eyes treated with brolucizumab indicated SRF presence than aflibercept after the loading doses. These beneficial effects of brolucizumab were observed with significant (p < 0.0001) lesser number of injections (1.8 ± 1.1 vs. 3.8 ± 1.5) from week 12 to week 48. Moreover, the probability of no injections after the loading doses was significantly higher with brolucizumab compared to aflibercept indicating prolonged injection-free intervals. The average ocular side effects were comparable in the two groups. One adverse event of severe vitritis requiring treatment with oral steroids occurred in Brolucizumab group, while no such event occurred in Aflibercept group.

**Conclusion:**

The results of the present study suggest non-inferiority of brolucizumab PRN regimen to aflibercept PRN regimen in treatment naïve nAMD Indian patients while achieving longer inter-injection intervals.

*Trial registration *Clinical Trial Registration of India (CTRI/2021/06/034415). Registered 03 March, 2021, http://ctri.nic.in/Clinicaltrials/pmaindet2.php?trialid=54328&EncHid=&userName =

## Background

Neovascular age-related macular degeneration (nAMD) is a chronic degenerative eye disorder affecting the macular region of retina and leads to gradual vision loss. The disease is associated with the growth of abnormal blood vessels from the choroid in the usually avascular retinal regions, thus leading to choroidal neovascularization (CNV), also known as macular neovascularisation (MNV) [1]. The disease accounts for the third leading cause of blindness (8.7%) globally. It is projected to affect 288 million people worldwide by 2040 as the aging population rises [2]. The prevalence of AMD is comparatively higher in western countries, predominantly European regions, than in India. But it adds to a significant visual concern owing to the large population base [3, 4]. There is a paucity of prevalence data of late-stage nAMD in India. Few population studies report it ranging from 0.11 to 1.2% [5–7]. Late-stage AMD is associated with poor prognosis in the elderly if left untreated [8].

Anti-vascular endothelial growth factor (anti-VEGF) therapies have been proven to be a milestone in nAMD treatment in the last 15 years [9]. Since then, several anti-VEGF agents have been used clinically to treat AMD. Conventional anti-VEGF agents available are Bevacizumab, Ranibizumab, Aflibercept, and Brolucizumab, with brolucizumab being the latest addition [10]. At present, ranibizumab and aflibercept serve as first-line agents for the treatment of nAMD. In addition, ranibizumab is FDA approved at a dosing interval of 4 weeks while aflibercept at 8 weeks after 3 loading doses for managing nAMD [9]. However, although found effective, these drugs have limitations, such as poor compliance, frequent dosing injections, and economic burden. Therefore, long-acting anti-VEGF agents that may prolong the dosing interval may help improve the efficacy and adherence [9, 11].

Brolucizumab, a humanized, single-chain antibody fragment against VEGF-A, was approved for the treatment of nAMD in 2019 by the United States Food and Drug Association (USFDA) [10, 12, 13]. The phase 3 trials evaluating brolucizumab (6 mg, q12w), HAWK, and HARRIER trials showed comparable best-corrected visual acuity (BCVA) gains with fixed q8w aflibercept therapy. Furthermore, a significant number of patients maintained the 12-week dosing interval without any substantial increase in the adverse event profile [14]. Thus, it offers a promising possibility of increasing the duration of hospital visits for patients with nAMD. However, most of the patient population in the HAWK and HARRIER trials were outside India. Since the AMD burden and treatment may suffer regional and genetic disparity [4, 15], studies indicating its efficacy and safety in the Indian population are imperative.

Thus, the objective of this study was to evaluate the efficacy, duration of injection intervals, and safety of brolucizumab in the Indian population compared to aflibercept.

## Methods

### Study design

The present study was a prospective, randomized, single-centre, single-blinded, two-arm comparative study assessing the efficacy of brolucizumab compared to aflibercept in treatment-naïve nAMD patients (Clinical trial registration of India number CTRI/2021/06/034415). The local Institutional Ethics Committee of Army Hospital Research & Referral, Delhi Cantt, approved the protocol (Approval number 135/2020, dated Dec 7, 2020). The trial was conducted compliant with the principles of the Declaration of Helsinki. Prior to recruitment in the study, written informed consent was obtained from all the participants. The study was carried out at Army Hospital Research & Referral, Delhi Cantt, a tertiary care centre in New Delhi, India.

### Study participants

Consecutive patients of untreated active CNV due to nAMD visiting our centre were recruited between December 2020 and February 2021, with no prior history of receiving any approved treatment for nAMD. They were assessed for inclusion and exclusion criteria (Table 1). At screening, all patients underwent detailed visual assessment with measurement of BCVA and a complete ophthalmic examination including slit-lamp examination, intraocular pressure measurement, and dilated fundus examination. In addition, all patients underwent Optical Coherence Tomography (OCT) imaging (Cirrus 5000, Carl Zeiss Meditec, Germany) to confirm the presence of MNV, SRF, and IRF. Further, patients showing active MNV underwent fundus fluorescein angiography (FFA) and indocyanine green angiography (ICGA) (FF-450, Carl Zeiss Meditec, Germany). The entire clinical evaluation and multimodal imaging interpretation were made by two experienced vitreoretinal surgeons of our institute. All the demographic variables were collected at the baseline visit.

**Table 1:** Inclusion and exclusion criteria for participation in the study

	Inclusion criteria
	Age more than 50 years
	Typical morphology of age-related macular degeneration (AMD)
	Lesion affecting the central subfield (1 mm around the centre)
	Active choroidal neovascularization comprising more than 50% of the total lesion area
	Intraretinal or Subretinal Fluid in central subfield
	Best-corrected visual acuity between 20/32 to 20/400

	Exclusion criteria
	Any fibrosis or geographical atrophy of central subfield
	Any intra or periocular infection or inflammation
	If the patient had received any approved treatment for neovascular AMD other than micronutrient supplementation
	Any concurrent intraocular disease like diabetic retinopathy
	History of drug sensitivity/ allergic reactions to research interventions
	Stroke or myocardial infarction in the 90 days preceding to baseline visit

### Randomization and treatment

The eyes of the nAMD patients were randomized 1:1 to either Group 1, which was brolucizumab arm (6 mg/50 µL, Pagenax, Novartis India Ltd, Mumbai, India) or Group 2, which was aflibercept arm (2 mg/50 µL, Eylea, Bayer, India) using randomization tables. The interventional treatment was provided as intravitreal (IVT) injections of the two drugs in the affected eyes under topical anaesthesia in aseptic conditions. The treatment regimen involved initial loading i.e., 3 loading doses, followed by individualized *pro re nata* (PRN) regimen. Further the treatment was provided based on the signs of active CNV upon OCT and BCVA assessment of each patient.

The interventional PRN treatment was initiated by administering the loading doses (brolucizumab and aflibercept) at weeks 0 (baseline), 4, and 8. Following these 03 four weekly doses, all patients were examined at 4-weekly intervals (week 12–48) for disease activity assessment by a masked investigator. All patients underwent BCVA measurement, clinical ophthalmic examination, and macular OCT evaluation at each visit. At each visit, the patients meeting the retreatment criteria (Table 2) were given the additional respective injection at the same dose; Group 1 received IVT brolucizumab 6.0 mg, and Group 2 received aflibercept 2.0 mg. The patients were blinded to the provided treatment. Further, the masked investigator decided to re-inject at each visit based on the BCVA and OCT macula interpretation supplied by the vitreoretinal surgeons. The results were then analysed by a reading team that was blinded to the treatment administered to the patient.

**Table 2:** Retreatment criteria for patients requiring additional respective injections (Brolucizumab or Aflibercept) at each visit

Retreatment criteria
BCVA decrease by 0.10 from BCVA gained at 12-week review or last visit
OCT CMT increase by 75 µm from CMT attained at 12-week review
The appearance of new IRF spaces in central 1.5 mm, i.e. 750 µm from the foveal centre
The appearance of new SRF space in central 1.5 mm, i.e. 750 µm from the foveal centre
Increase in SRF height from the status achieved at 12-week review

*BCVA* Best-corrected visual acuity, *OCT* Optical coherence tomography, *CMT* Central macular thickness, *IRF* intraretinal fluid, *SRF* subretinal fluid

### Outcome measurements

The primary efficacy parameters were changes in BCVA and central macular thickness (CMT) at weeks 12 and 48. The BCVA measurements were conducted in both eyes at screening and every 4 weeks after that, using the logMAR (Logarithm of the Minimum Angle of Resolution) chart (I Chart HD, Appasamy Associates, Chennai, India). It was conducted by a team of certified optometrists masked to the study and reported up to the second unit decimal in logMAR. The CMT was measured using the Cirrus 5000 HD OCT in both eyes at screening and every 4 weeks after that. The tracking function was used to measure maximum readings through the same central point at all visits. The CMT assessment was conducted by two vitreoretinal surgeons to ensure the accuracy of the findings. The secondary efficacy parameters involved the review of the presence or absence of IRF, SRF or subretinal hyper-reflective material (SHRM) in the study eye at each visit. Besides, the average number of injections required for both the drugs and the average injection-free interval were also assessed. The same OCT imaging system was used throughout the study period at each visit to maintain consistency.

Additionally, safety evaluations involved measuring ocular adverse events (AE) throughout the treatment period. The AE were scored as 0 (no adverse events), 1 (subconjunctival haemorrhage and pain not requiring oral analgesic), 2 (anterior chamber [AC] cells and flare less than or equal to two with no circumciliary congestion, or ocular pain requiring oral analgesic), 3 (AC cells and flare more than two, circumciliary congestion, vitritis grade 1, and patient not requiring oral steroids for management) and 4 (Hypopyon in AC, synechiae formation, vitritis more than grade 2, optic disc oedema/hyperaemia, retinal vascular sheathing/haemorrhages in retina in addition to pre-existing CNV).

### Endpoints and statistical analysis

The study endpoints included BCVA change from baseline to week 12 and baseline to week 48, absolute values and mean reduction in CMT from baseline to week 48, status of IRF, SRF and SHRM up to week 48, the average number of injections required, average injection-free interval and incidence of any ocular AE.

The sample size for the two arms was calculated considering 80% power and significance level of 0.05 (5%), disease prevalence of 1.47%, mean change in brolucizumab arm in previous studies at 5.9 letters and aflibercept arm at 5.3 letters, standard deviation (SD) of 14.79 for both arms, the margin of 9 letters, and attrition of 10%.

The statistical analysis was performed using R software (version 4.1.2) for Windows. The numerical variables were summarized descriptively as mean, median, standard deviation, minimum and maximum using the number of subjects in each treatment group. Categorical variables were summarized using count and percentage. BCVA was measured using Snellen's chart and converted into logMAR values in decimal units. As appropriate, the between-group comparisons for categorical variables were done using chi-square test or Fisher's exact test. While the numerical variables were compared using paired *t*-test and two-sample *t*-test for within-group and between-group comparisons, respectively. Kaplan–Meier method was conducted for the survival analysis on the injection-free duration. The subjects who did not take any injection at 48 weeks were censored. The log-rank test was used to compare the overall injection-free duration between the two treatment arms. The interquartile range was also provided for the injection-free duration. p-values < 0.05 were considered statistically significant.

## Results

### Study participants

**Fig. 1:**
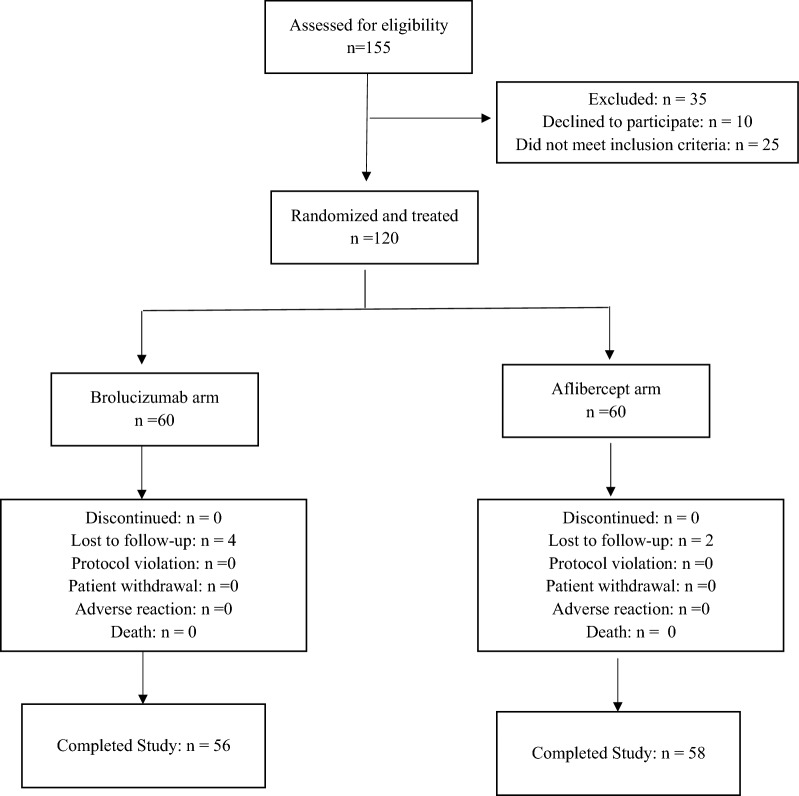
Flowchart of participants

During the study period, March 2021–February 2022, a total of 155 patients were assessed for eligibility, and 120 were found to be eligible. These patients were enrolled and randomized to receive either brolucizumab or aflibercept treatment. (60 eyes in each group, Fig. 1). Of the 120 patients randomized to treatment, 06 patients were lost to follow-up (04 in brolucizumab and 02 in aflibercept arm). Thus, the final analysis population included 56 patients in the brolucizumab and 58 patients in the aflibercept arm.

The demographic and baseline characteristics of the patients were balanced between both arms, as depicted in Table 3. The baseline BCVA was 0.84 ± 0.32 and 0.87 ± 0.27 in the brolucizumab and aflibercept arms, respectively. The absolute CMT values were balanced among both the arms (355.0 ± 89.7 in the brolucizumab arm and 365.5 ± 55.8 in the aflibercept arm). At baseline, IRF was present in 82.1% and 75.9%, SRF in 82.1% and 82.8%, while SHRM in 42.9% and 53.4% of patients in brolucizumab and aflibercept arm, respectively.

**Table 3:** The demographics and baseline characteristics of the patients

Characteristics	Brolucizumab N = 56	Aflibercept N = 58	P value
Age, years			
Mean ± SD	72.05 ± 11.03	70.17 ± 9.02	0.32
Median	72.00	70.5	
Min, max	51.00, 91.00	54.00, 91.00	
Sex, n (%)			0.17
Male	37 (66.1)	30 (51.7)	
Female	19 (33.9)	28 (48.3)	
LogMAR BCVA			
Mean ± SD	0.84 ± 0.32	0.87 ± 0.27	0.63
Min, max	0.28, 1.42	0.26, 1.30	
CMT (µm)			
Mean ± SD	355 ± 89.7	365.5 ± 55.78	0.46
Min, max	211.00, 598.00	265.00, 566.00	
Presence of IRF n (%)	46 (82.1)	44 (75.9)	0.55
Presence of SRF n (%)	46 (82.1)	48 (82.8)	> 0.99
Presence of SHRM n (%)	24 (42.9)	31 (53.4)	0.35

Data presented as mean ± standard deviation or n (%)

*BCVA* Best corrected visual acuity, *CMT* Central macular thickness, *IRF* intraretinal fluid, *SRF* subretinal fluid, *SHRM* subretinal hyper-reflective material, *SD* standard deviation

*p-value < 0.05 for the t-test considered significant (none of the t-test found significance)

^#^p-values < 0.05 for the chi-square test considered for significant correlation (none of the chi-square test found correlation)

### Efficacy parameters

**Fig. 2:**
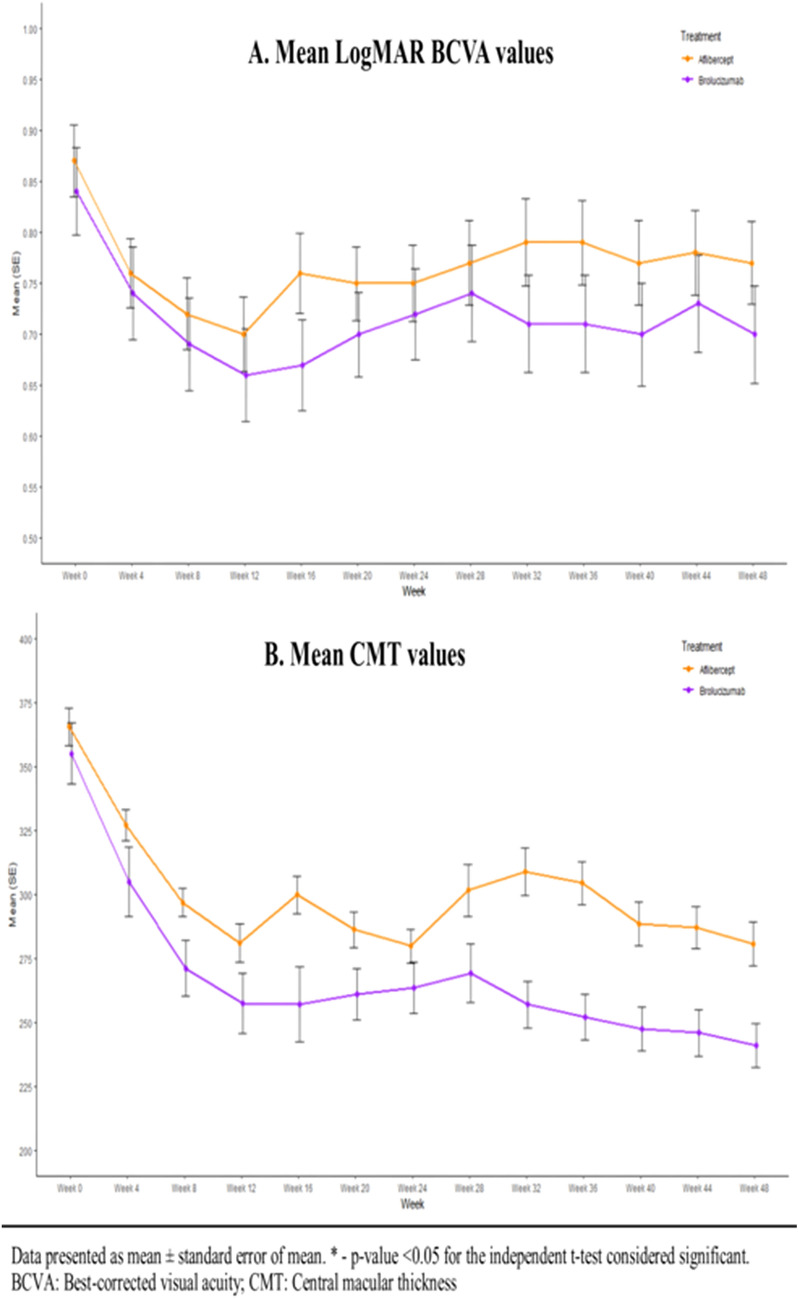
**A** The mean LogMAR BCVA values from baseline visit to week 48 visit for the patients treated with brolucizumab and aflibercept. **B** The mean CMT values from baseline to week 48 for the patients treated with brolucizumab and aflibercept

The significant mean BCVA change was observed in both brolucizumab and aflibercept arms, respectively, from baseline to week 12 and from baseline to week 48. The mean change in logMAR BCVA from baseline to week 48 was −0.13 ± 0.21 for brolucizumab, and −0.10 ± 0.15 for aflibercept, respectively, with a difference of 0.03 [80% confidence interval (CI), −0.04 to 0.10] between the arms. At week 12, the mean change in logMAR BCVA from baseline was −0.18 ± 0.2 and −0.17 ± 0.15 for the brolucizumab and aflibercept arm. The mean change in logMAR BCVA at week 12 (p = 0.81) and 48 (p = 0.37) was found to be non-significant among both the groups. Further, these results indicated significant visual gains in BCVA for brolucizumab (p < 0.0001) as well as aflibercept (p < 0.0001). Figure 2A depicts the mean logMAR estimates for BCVA from baseline (week 0) to week 48. These results demonstrate that efficacy of brolucizumab is similar to aflibercept at weeks 12 and 48 without any statistically significant difference in BCVA.

The mean estimates for CMT from baseline (week 0) to week 48 are presented in Fig. 2B. The primary endpoint of mean CMT change from week 0 to week 48 was −112.59 ± 81.23 µm and −86.38 ± 71.82 µm for brolucizumab and aflibercept, respectively with a difference of −26.21 (80% CI, −2.89 to 55.31). At week 12, the same was observed to be −97.34 ± 84.13 µm and −84.34 ± 71.51 µm, respectively. There was no significant difference observed between the two groups at week 12 as well as week 48, which indicates non-inferiority of brolucizumab compared to aflibercept. Also, there was a significant reduction in CMT from baseline to week 48 in both groups (p < 0.0001). These results in Fig. 2B demonstrate that efficacy of brolucizumab is similar to aflibercept at weeks 12 and 48 without any statistically significant difference in CMT.

**Fig. 3:**
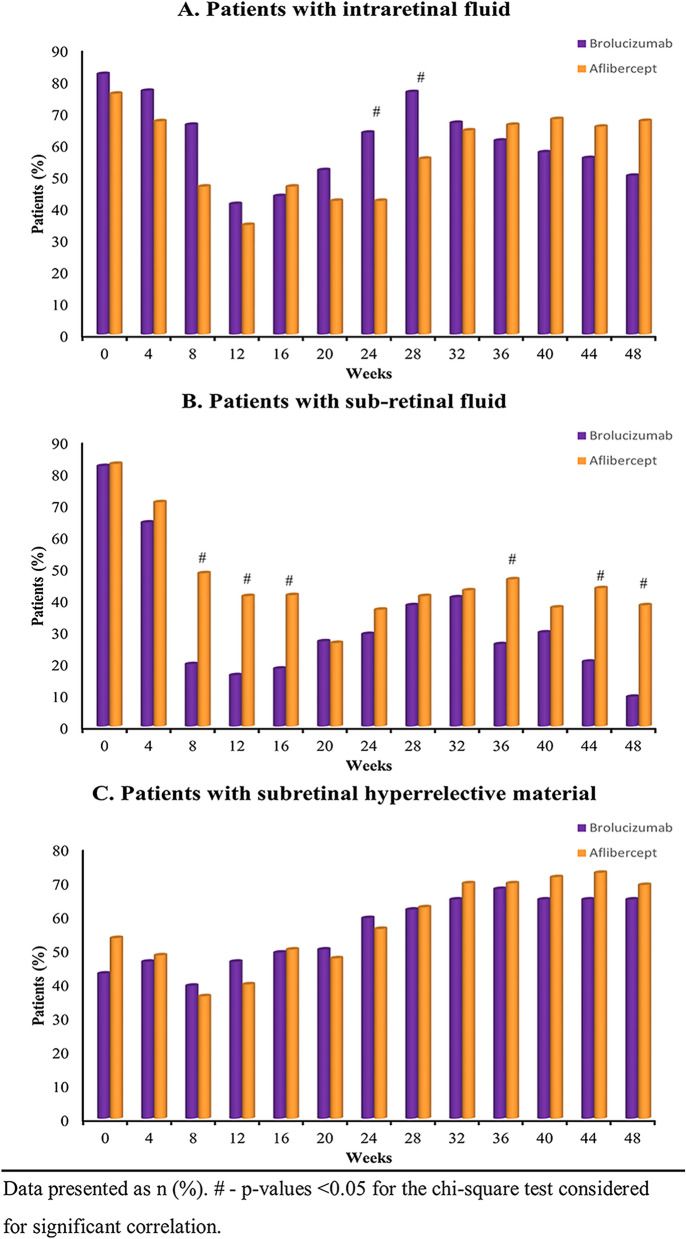
**A** Proportion of patients with presence of intraretinal fluid from baseline visit to week 48 visit. **B** Proportion of patients with presence of subretinal fluid from baseline visit to week 48 visit. **C** Proportion of patients with presence of subretinal hyperreflective material from baseline visit to week 48 visit

Figure 3 depicts the analysis of the presence or absence of IRF, SRF and SHRM in eyes treated with brolucizumab and aflibercept throughout the study period. The percentage of eyes with IRF for brolucizumab and aflibercept arm, respectively, at week 12 was 41.1% and 34.5% (p = 0.59) and at week 48 was 50.0% and 67.3% (p = 0.10), indicating non-inferiority of brolucizumab (Fig. 3A). Furthermore, the percentage of eyes with SRF for brolucizumab and aflibercept arm, respectively, at week 12 was 16.1% and 41.4% (p = 0.006) and at week 48 was 9.3% and 38.2% (p = 0.0009), indicating a significant beneficial effect of brolucizumab compared to aflibercept (Fig. 3B). Further, at week 12, SHRM was observed in 46.4% and 39.7% (p = 0.59) of patients treated with brolucizumab and aflibercept, respectively, which was 64.8% and 69.1% at week 48 (p = 0.79) (Fig. 3C). This indicated no significant differences between both the groups.

**Fig. 4:**
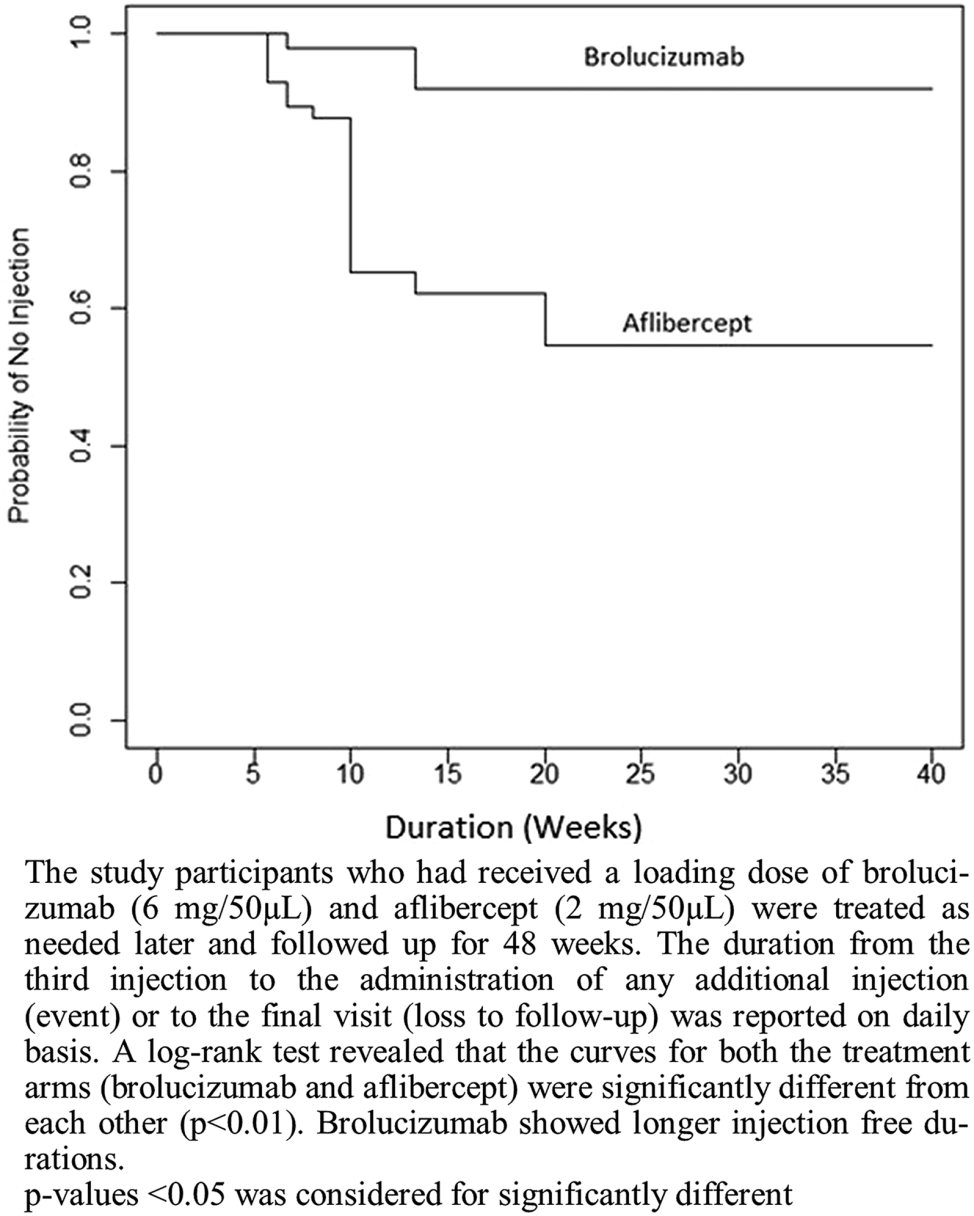
Kaplan–Meier curve for injection-free duration after the loading doses of brolucizumab and aflibercept

Furthermore, the mean number of injections required from week 12 to week 48 after the 3 loading doses in the brolucizumab arm was 1.8 ± 1.1 injections which were significantly less (p < 0.0001) compared to the aflibercept arm (3.8 ± 1.5 injections). The injection-free duration was computed for both treatments. The survival analysis was conducted to compare the probability of no injection between the two treatments, viz., brolucizumab and aflibercept. The median probability of no injection for brolucizumab was observed to be 2.9 (2.9, 5.7) weeks, while that of aflibercept was 1.4 (1.4, 1.9) weeks. The Kaplan–Meier curve for the probability of no injections was found to be significantly different (p < 0.01) between the two arms (Fig. 4), indicating that long dosing intervals were achieved successfully with brolucizumab. Table 4 shows the distribution of patients according to the additional injections required from week 12 to week 48.

**Table 4:** The distribution of patients according to the additional injections required during the week 12 to week 48

Treatment	Between 12 and 16 weeks	Between 20 and 24 weeks	Between 28 and 32 weeks	Between 36 and 40 weeks	Between 44 and 48 weeks
Brolucizumab (n = 56)	3 (5.4%)	17 (30.4%)	3 (5.4%)	1 (1.8%)	2 (3.6%)
Aflibercept (n = 58)	23 (39.7%)	21 (36.2%)	13 (22.4%)	12 (20.7%)	8 (13.8%)

The inter-injection interval after receiving the first three loading doses in both arms have been tabulated in Table 5. The number of injections in Brolucizumab arm after week 8 was 94, compared to 180 in Aflibercept arm. The percentage of injections administered at an interval of 4 weeks, 8 weeks, 12 weeks, 16 weeks and 18 weeks was 17%, 7.5%, 19.2%, 24.5% and 13.8% in Brolucizumab group compared to 43.9%, 21.7%, 20.6%, 7.2% and 5% in Aflibercept group. Five patients in Brolucizumab group never received another injection after week 8 compared to one patient in Aflibercept group.

**Table 5:** The frequency of patients achieving different inter-injection intervals after receiving the third loading dose at week 8 in the two groups

Treatment	Interval of 4 weeks from last injection	Interval of 8 weeks from last injection	Interval of 12 weeks from last injection	Interval of 16 weeks from last injection	Interval of 20 weeks from last injection	Interval of 24 weeks from last injection	Interval of 28 weeks from last injection	Interval of 32 weeks from last injection	Interval of 36 weeks from last injection	NO injection after week 8
Brolucizumab (94 injections after week 8)	16	7	18	23	13	11	4	1	1	5
Aflibercept(180 injections after week 8)	79	39	37	13	9	2	1	0	0	1

**Fig. 5:**
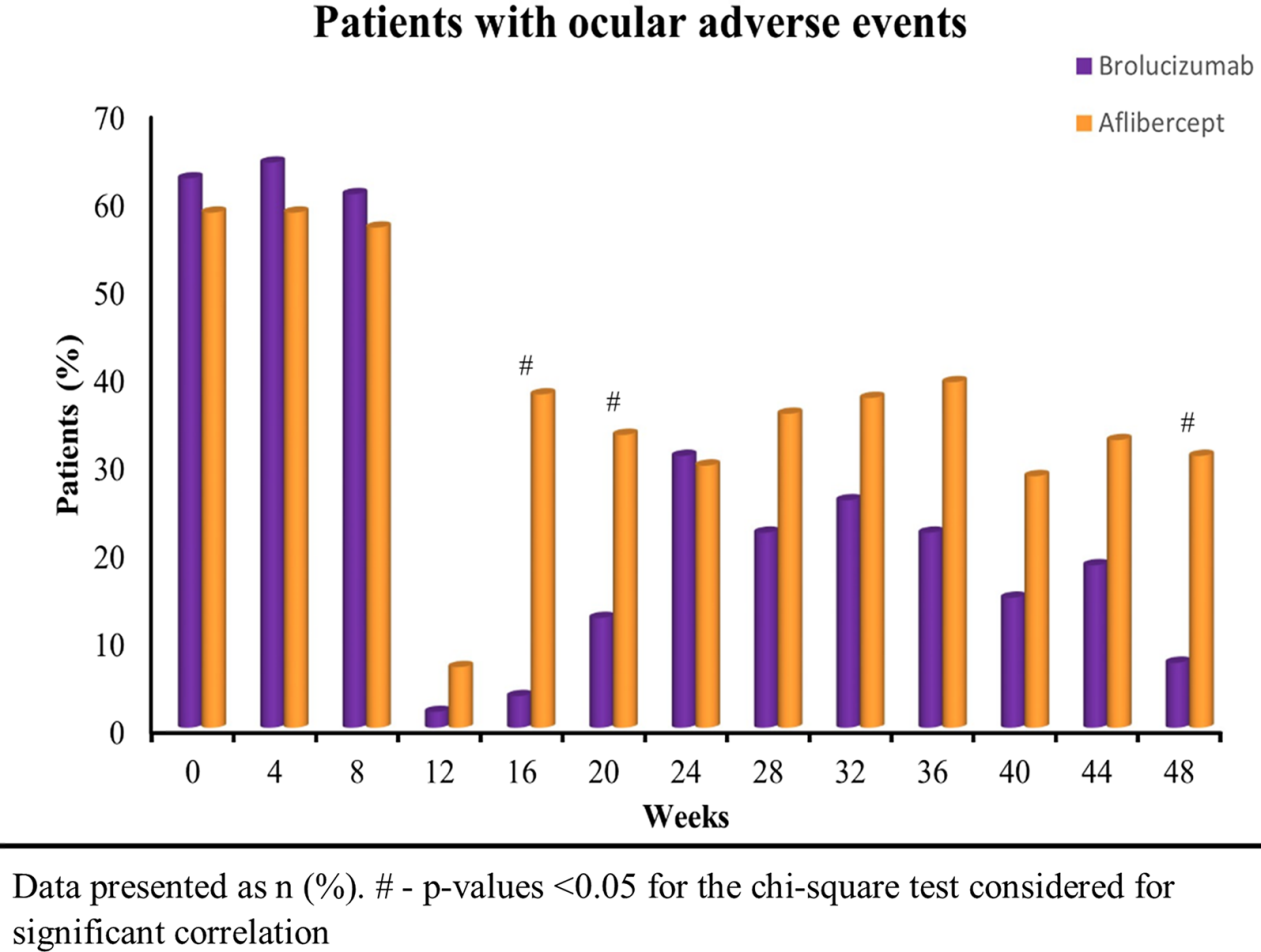
The frequency of ocular adverse events observed in brolucizumab and aflibercept arm throughout the study period of 48 weeks

The frequency of AE improved significantly from baseline (62.5% in brolucizumab and 58.6% in aflibercept arm) to week 48 with 7.4% and 30.9% in the brolucizumab and aflibercept arm, respectively (Fig. 5). Overall, brolucizumab was well tolerated with limited ocular AE. Further, the frequency of AE was significantly less in brolucizumab arm than aflibercept arm at week 16 (p < 0.0001), 20 (p = 0.016) and 48 (p = 0.003) (Fig. 5). Table 6 represents the total mean scores of the AE per injection observed in each group at each visit. The mean side effect score per injection was significantly lower (p = 0.002) in the brolucizumab arm (0.07 ± 0.26) compared to the aflibercept arm (0.31 ± 0.47) at week 48 (Table 6). The most common AE was subconjunctival haemorrhage. Other observed ocular AE included presence of AC cells and flare, circumciliary congestion and one instance of severe vitritis. No presence of hypopyon in AC, synechiae formation, optic disc oedema/hyperaemia, retinal vascular sheathing or haemorrhages in the retina in addition to pre-existing MNV were noted during the study. No deaths were observed in any of the treatment arms. Also, none of the patients in both arms discontinued the treatment owing to AEs.

**Table 6:** The total mean score of the ocular adverse events per injection observed in each group

Week/Visit	Total mean score of the adverse events		p value
	Brolucizumab arm n = 56	Aflibercept arm n = 58	
Week 0	0.73 ± 0.70	0.64 ± 0.58	0.44
Week 4	0.68 ± 0.54	0.66 ± 0.61	0.83
Week 8	0.80 ± 0.82	0.69 ± 0.71	0.43
Week 12	0.02 ± 0.13	0.07 ± 0.26	0.18
Week 16	0.04 ± 0.19	0.43 ± 0.62	< 0.0001*
Week 20	0.18 ± 0.54	0.40 ± 0.62	0.04*
Week 24	0.40 ± 0.68	0.35 ± 0.58	0.68
Week 28	0.26 ± 0.56	0.43 ± 0.63	0.14
Week 32	0.33 ± 0.67	0.45 ± 0.63	0.36
Week 36	0.24 ± 0.47	0.48 ± 0.66	0.03*
Week 40	0.20 ± 0.63	0.32 ± 0.54	0.30
Week 44	0.20 ± 0.45	0.33 ± 0.47	0.17
Week 48	0.07 ± 0.26	0.31 ± 0.47	0.002*

Data presented as mean ± standard deviation

*p-value < 0.05 for the t-test considered significant

Score 0: No adverse events; Score 1: Subconjunctival haemorrhage, Pain not requiring oral NSAIDs; Score 2: Anterior chamber (AC) cells and flare less than or equal to two. No circumciliary congestion. Ocular pain requiring oral NSAID; Score 3: AC cells and flare more than two, circumciliary congestion, vitritis grade 1. Patient not requiring oral steroids for management; Score 4: Hypopyon in AC, synechiae formation, vitritis more than grade 2, optic disc oedema/hyperaemia, retinal vascular sheathing/haemorrhages in retina in addition to pre-existing choroidal neovascularization.

Only one patient in Brolucizumab group showed severe vitritis requiring treatment with oral steroids while no such event occurred in Aflibercept group. This patient had previously received 05 doses of Brolucizumab at 0, 4, 8, 16 and 28 weeks and developed this vitritis after the sixth dose of Brolucizumab at 40th week. The patient responded to treatment within two weeks and BCVA improved from 0.98 at week 40 (pre-injection) to 0.88 at week 48. The adverse events score during the entire study is highlighted in Table 7. Though the number of AEs is more in Aflibercept group, it is primarily due to a greater number of Aflibercept injections given after week 8 (180 injections) compared to Brolucizumab group (94 injections). The serious AEs (score 3 & 4) were more in Brolucizumab group. However it is pertinent to mention that the score 3 AEs responded to topical drugs alone and continued receiving further treatment with Brolucizumab during the study.

**Table 7:** The frequency of AE scores in the two groups

Score of AE	Brolucizumab group	Aflibercept group
Total number of injections in all patients	262	354
Total number of AE reported	219	314
Score 1	195 (89.0%)	277 (88.2%)
Score 2	14 (6.4%)	35 (11.2%)
Score 3	9 (4.1%)	2 (0.64%)
Score 4	1 (0.46%)	0

## Discussion

The HAWK and HARRIER phase 3 trials have shown the non-inferiority of brolucizumab against aflibercept, with more than 50% of patients with nAMD maintaining the BCVA gains on q12w dosing interval throughout the study period of 48–96 weeks [14, 15]. In addition, brolucizumab has shown effectiveness in improving visual acuity in several studies [13–20]. However, the treatment for nAMD may respond differently in patients at different stages of the disease [21] as well as the treatment may differ in patients with diverse ethnicity and genetic make-up [4, 22, 23]. Thus, it is imperative to pool the efficacy data of brolucizumab from different populations belonging to various geographical locations and ethnicity. Unfortunately, the patient population in the HAWK and HARRIER studies did not include Indian patients. Thus, our study intended to evaluate the efficacy and non-inferiority of brolucizumab versus aflibercept in the Indian population.

Our study demonstrates the efficacy of brolucizumab to maintain the BCVA gains among a significant number of nAMD patients on a longer dosing interval over 48 weeks that were comparable to aflibercept therapy with shorter dosing intervals. The inter injection frequency data presented in Table 5 shows the achievement of injection free intervals up to 12 or more weeks in 71 out of 94 instances (75.5%), of re-injections after week 8, in the Brolucizumab group. The same data for Aflibercept was 62 eyes out of 180 (34.4%) achieving more than or equal to 12-week inter-injection interval and 101 out of 180 (56.1%) achieving more than or equal to 8-week inter-injection interval.

The measurement of BCVA changes can help evaluate the treatment’s efficacy in patients with nAMD [24]. Significant improvements were observed in the functional outcomes, i.e., BCVA gains. The logMAR BCVA in Indian patients in our study was significantly improved from 0.84 ± 0.32 at baseline to 0.70 ± 0.35 at week 48. The improvement was comparable to aflibercept demonstrating non-inferiority of brolucizumab as a treatment option for nAMD in Indian patients.

Further, anatomical outcomes assessed by OCT, such as CMT changes and the presence of retinal fluid (IRF, SRF, and SHRM), are important indicators of disease activity in nAMD patients [14, 23]. In our study, significant improvement was observed in CMT from baseline to week 48 in eyes treated with both the drugs. However, our results indicate that changes in CMT more significantly favoured brolucizumab over aflibercept. Furthermore, the retinal fluid resolution observed in patients treated with brolucizumab was better than aflibercept. The greater IRF resolution in brolucizumab treated patients was evident by weeks 24 and 28. At both time points, the majority of the brolucizumab patients were on a 12-week dosing interval compared to an 8-week dosing interval for aflibercept treated patients. Additionally, it has been shown that individuals with residual SRF at the end of the loading doses may have a negative impact on BCVA gains [15, 23]. Our results indicate fewer eyes treated with brolucizumab with SRF presence than aflibercept after the loading doses, i.e., at weeks 8, 12 and 16. This benefit was also maintained at weeks 44 and 48. These results corroborate previous studies [15, 17, 19, 20]. The SHRM resolution was comparable for both brolucizumab and aflibercept treated eyes. Any retinal fluid presence in the eyes suggests sub-optimal disease control [23, 25, 26]. Thus, improvement in retinal fluid resolution in brolucizumab treated eyes demonstrates an effective anti-VEGF therapy for nAMD Indian patients in clinical practice.

Furthermore, the number of injections required from week 12 to week 48 after 03 monthly doses were significantly lower for patients in the brolucizumab arm than the aflibercept arm. The average injection-free interval as computed by the probability of no injections between two intervals in the brolucizumab arm was also significantly higher compared to the aflibercept arm (Fig. 4). These results endorse the results of the study conducted by Dugel et al. (2017) with respect to the number of additional unscheduled treatments required. A significantly less proportion of patients required additional unscheduled treatments in the brolucizumab groups than the aflibercept group [27]. Also, similar results of improving the treatment interval were obtained by Rave et al. (2021) in real life clinical setting [28]. Thus, the improved injection-free interval can help to improve patient compliance as it may reduce the burden of treatment and monitoring visits. Also, the proportion of patients who required any additional injections was less in the brolucizumab arm compared to the aflibercept arm (Table 4). These results indicate the potentiality of brolucizumab in maintaining disease stability. Moreover, the safety profile of brolucizumab in our study was comparable to the safety profile found in the HAWK and HARRIER study as well as to aflibercept [14]. The average ocular side effect score at the end of the study was significantly better with brolucizumab than aflibercept.

A specific note on the effect of both the drugs on polypoidal choroidal vasculopathy (PCV) has not been made as the same was not incorporated in the study design. However, no gross deviation in the outcome parameters was noted in the subset of PCV patients during the study.

Our study suffers from the limitation of study duration being small when comparing the natural course of nAMD which may span few decades. A longer prospective study will bring out the long-term effects of these drugs on yet unknown dimensions of disease outcomes. The present study parameters also did not include a subset selection of type of MNV (Type 1, Type 2 or Type 3) or Polypoidal Choroidal Vasculopathy (PCV) for their specific response to the new molecule, i.e. brolucizumab.

## Conclusions

In conclusion, significantly better BCVA gains, fluid resolution along with minimal ocular adverse events over 48 weeks were observed in eyes treated with brolucizumab monotherapy. The benefits observed with brolucizumab PRN therapy were non-inferior to aflibercept PRN therapy in patients with nAMD. The inter-injection interval achieved by Brolucizumab was higher than Aflibercept. This analysis suggests that brolucizumab could help alleviate the treatment and hospital visit burden associated with nAMD in Indian patients.

## Data Availability

The datasets used and/or analysed during the current study will be available from the corresponding author on reasonable request.
